# What Influences Consumers’ Intention to Purchase Innovative Products: Evidence From China

**DOI:** 10.3389/fpsyg.2022.838244

**Published:** 2022-03-30

**Authors:** Jie Li, Fan Guo, Jialin Xu, Zucheng Yu

**Affiliations:** ^1^School of Management, Shanghai University, Shanghai, China; ^2^International Business School Suzhou, Xi’an Jiaotong-Liverpool University, Suzhou, China; ^3^School of International Relations and Public Affairs, Shanghai International Studies University, Shanghai, China

**Keywords:** risk propensity, cognitive legitimacy, purchase intention, the theory of planned behavior, Chinese consumer

## Abstract

Drawing on the theory of planned behavior, we investigate the legitimacy of platform governance and whether consumers with greater ranges of risk propensity are more likely to purchase innovative products. This study develops a moderated mediation model involving risk propensity, cognitive legitimacy, purchase intention and perceived benefit. To examine our hypotheses, we conducted a survey of 315 consumers from Shanghai, China. The results reveal that risk propensity is positively related to consumers’ purchase intentions, in which cognitive legitimacy plays a mediating role. Furthermore, the interaction suggests that perceived benefit moderates the relationship between risk propensity and cognitive legitimacy.

## Introduction

With the development of the knowledge economy and the improvement of digitization, emerging markets have gradually become the main growth engine in the business environment (Cahen et al., 2016). This business environment requires enterprises to constantly develop innovative products to meet market demand. Whether to launch innovative products has become an important force determining the sustained development of enterprises (Cooper, 2000). For example, ByteDance’s TikTok and other products have become very popular social platforms at home and abroad. We take social platforms as an example. With the rapid growth of platform traffic, short videos have become a new mainstream information carrier in globalization, which leads to changes in social networking, e-commerce and other industries. Considering this background, the healthy development of technology-based enterprises and the importance of platform governance have attracted a growing amount of attention from academia (Onetti et al., 2012).

Some scholars have discussed the social utility of innovative products. Innovative products are regarded as important factors in promoting employment and technology change (Almus and Nerlinger, 1999; Zhao et al., 2019). Compared with traditional products, the production cycle of innovative products is shorter, and the market share is occupied faster (Oviatt and McDougall, 1994; Knight and Kim, 2009; Onetti et al., 2012). On the other hand, as the market matures and consumers become more selective about products, competition for innovative products will be more fierce (Yamashina et al., 2002). In this context, scholars have studied the platform governance and market performance of innovative products. In the research on the market performance of innovative products, the function and appearance of products designed by consumers’ demand will influence the market performance of products through consumers’ purchase tendency (Gorwa, 2019). In view of the important role of innovative products and platform governance in the market economy, there is a growing need for research into innovative product purchase behaviors.

According to Bagozzi and Burnkrant (1979), purchase intention, different from purchase desire, is regarded as consumers’ subjective tendency to pay for the products or services. In our study, purchase intention is a conscious effort by consumers to choose products or services, which may be generated when the impression or attitude given to consumers meets their expectations (Spears and Singh, 2004). Purchase intention can predict purchase behavior well. In view of the significance of purchase intention, researchers have devoted considerable effort to investigate several factors that influence consumers’ purchase intention, including product information, trust, cultural differences, perceived quality and perceived risk (Chang and Wildt, 1994; Bian and Forsythe, 2012; Hajli et al., 2017).

In the international marketing management literature, purchase behavior is recognized as a risk. The choice to purchase new products always involves uncertainty about the consequences (Bauer, 1960). However, limited attention has been given to how risk affects consumers’ purchase intention. Given that risk is an important factor that influences consumer behavior, perceived risk may influence consumers’ purchase intention (Pelaez et al., 2019). Consequently, this article focuses on how risk propensity influences consumers’ purchase intention. Furthermore, firm behavior can have an impact on consumers’ purchase behavior (Creyer, 1997). However, previous studies generally ignored the impact of the relationship between firm behaviors and public psychological expectations on purchase intention (e.g., platform legitimacy). Indeed, companies such as ByteDance, the owner of TikTok, have a “duality”—it is not just a business but a market, with an increasing number of complex stakeholders involved, making it harder to gain legitimacy (Fenwick et al., 2019). Taking the short video platform as an example, its stakeholders include not only its employees and shareholders but also the creators, users and advertisers on the platform and even competitors such as traditional media. In many cases, the interests of these stakeholders are contradictory or even conflicting with each other. The pursuit of legitimacy can make it easier for enterprises to attract consumers, thereby increasing product profits (Delmar and Shane, 2004). Therefore, we introduce perceived legitimacy of the platform as a crucial factor influencing whether consumers generate purchase intention.

**Figure 1:**
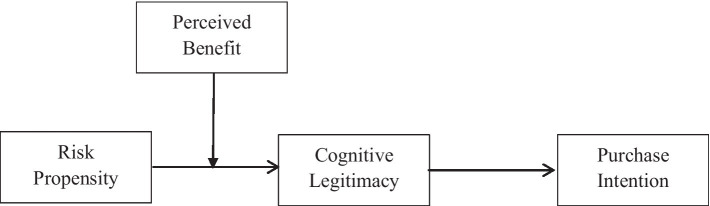
Hypothesized model of processes linking risk propensity and purchase intention.

The current research contributes to the literature on purchase intention from many aspects. First, it investigates the marketing management of innovative products, which is an overlooked area. Through a detailed explanation of the innovative product situation, the study helps enrich the research on purchase intention. Second, this study explores the impact of risk propensity on consumers’ purchase intention. Specifically, it proposes that cognitive legitimacy mediates the relationship mentioned above. The intermediary relationship not only enriches the literature on risk and purchase but also contributes to a better understanding of consumers’ attitudes toward organizational legitimacy. In addition, the findings of this study also have enterprise application value and provide a reference for promoting the purchase intention of consumers and the challenges faced by the legitimacy of management. Figure 1 shows the research model in detail.

## Hypothesis Development

### Theory of Planned Behavior

Ajzen (1991) first proposed the theory of planned behavior (TPB). TPB emphasizes that individuals can rationally decide whether to take action by systematically evaluating the available information and behavioral intention (Fishbein and Ajzen, 1977). Behavioral intention is the motivation of individual behavior, indicating that individuals have the tendency to take behavior (Ajzen, 1991) and are directly controlled by factors including perceived behavior, subjective norms and attitudes (Ajzen and Madden, 1986). In particular, attitude was first defined as the psychological tendency to like or dislike something or the cognitive and emotional tendency to something (Ajzen et al., 1982). With the development of theories, scholars define attitude as a state of learning and organizing with the help of experience from the perspective of psychology (Ivancevich, 2010). Subjective norms refer to one’s perceived social pressure from weighty individuals or groups to act in a particular way (Ajzen, 1991). In the field of marketing, we accept the definition of subjective norms, which are the views or opinions that consumers receive from the community when paying for a product or service (Ajzen, 1991; Klöckner, 2013). Previous studies have shown that it is precisely because of the pressure from others that subjective norms enhance or inhibit individual behavior (López-Mosquera et al., 2014). For example, when members of intimate groups hold a negative attitude toward a product or enterprise, consumers’ purchase intention will decrease. Subjective norms are closely involved in consumers’ purchase intentions. As an antecedent variable of behavioral intention, it influences behavior through willingness (Ha and Janda, 2012). Broadly speaking, purchase intention is generally considered to be the subjective probability that someone purchases a product (Dodds et al., 1991), which is regarded as the process of forming behavioral intention. Therefore, TPB can explain the process mechanism of purchase intention formation.

In recent years, scholars have explored the possible cognitive mechanism between behaviors and behavior intentions according to the theory of planned behavior. They finally proposed a possible cognitive mechanism—the implementation intention of behavior. There are two stages when an action occurs (Gollwitzer, 1999). The first stage is the motivational stage of behavior, and in this stage, behavioral motivation is influenced by the stages of perceived behavior, subjective norm, and attitude. The second stage is the implementation stage, which is related to the individual’s volition, and this stage is between intention and action. In the latter stage, the individual carries out these plans by making specific action plans, such as when and where to do what (Rise et al., 2003). Specific to our research, we argue that consumers are more likely to identify specific purchases when they maintain a positive perception of innovation (Ajzen et al., 1982). For example, consumers who perceive higher value in purchasing innovative products are more likely to purchase them.

### Risk Propensity and Purchase Intention

With the development of digitization, technology-based enterprises are increasingly confident in creating innovative products to meet consumer needs. For the consumers, every purchase is a risky behavior (Bauer, 1960). Risk is the most commonly considered factor in the purchase behavior of consumers. Previous studies have discussed perceived risk and user risk propensity. In a specific context, perceived risk is negatively related to consumer trust (Lim, 2003), and perceived quality indirectly affects the perceived value of consumers through the mediating role of perceived risk (Ledden et al., 2007). From the perspective of users’ risk propensity, the influence of risk propensity also affects attitudes toward resource acquisition (Wang et al., 2022) and consumers’ behavioral choices (Keil et al., 2000; Forlani et al., 2002).

Risk propensity is a personality trait that generally measures an individual’s preference for risk choices, which is stable over time (Fischhoff et al., 1983). Researchers argue that risk propensity is the assessment of whether it is worth taking risks to achieve better returns as well as one’s risk preference (Taylor and Dunnette, 1974; Sitkin and Pablo, 1992). Subdivided into the field of psychology and sociology, scholars point out that the relationship between risk propensity and consumer behavior mainly shows that consumers’ own risk propensity will affect their attention to policies. Consumers with low risk propensity tend to choose products with which they are familiar, depending on the fact that they are more influenced by external authoritative policies and reliable information (Khashe and Heydarian, 2016). The purpose is to reduce internal perceived risks and improve the correctness of purchase decisions.

In accordance with this perspective, we view risk propensity as one’s behavioral tendency, which is fashioned through an amassed trip of previous behaviors, varying by personality. However, people with excessive risk propensity are more likely to pursue risk-increasing techniques while improving their returns in the effect (Cho and Lee, 2006). Because of this excessive return, they are more likely to undertake volatile buying actions than those who do not like risk (Brockhaus, 1980). Therefore, we hypothesize the following:

*Hypothesis 1*: Risk propensity is positively related to consumers’ purchase intention.

### Mediating Role of Cognitive Legitimacy

The concepts of “legitimacy” vary, and we take Suchman (1995) and Scott’s (2013) point of view. They proposed that legitimacy refers to the action taken by an entity that is appropriate in social norms, values, beliefs, certainty or in a certain social system (Suchman, 1995). Generally, legitimacy includes regulatory legitimacy, normative legitimacy and cognitive legitimacy (Scott, 2013). It is worth noting that although legitimacy is a collective phenomenon, cognitive legitimacy is a comprehensive evaluation of individual actors (Bitektine and Haack, 2015). However, the role of individuals in organizational legitimacy and the ways in which individuals construct cognitive legitimacy perceptions have been underestimated (Deephouse and Suchman, 2008; Deephouse et al., 2017). This paper aims to study consumers’ recognition of innovation-oriented enterprise legitimacy, so cognitive legitimacy is adopted. Cognitive legitimacy refers to the behavior of enterprises in accordance with the general psychological cognition and expectations of the public.

The theory of planned behavior addresses that consumers’ purchase behavior is not only individual behavior but also influenced by subjective norms (Ajzen and Madden, 1986). Subjective norms are viewed as the perceived stress imposed by the public, such as neighbors, friends and many others (Ajzen and Driver, 1991). In other words, consumers will seek information from the public to improve their positive cognition before generating purchase intention, and this kind of socialized information can affect the individual’s internal psychological state (Li et al., 2018). Previous research demonstrates that when the product information provided by the enterprise is not clear, consumers will evaluate the product through the cognitive legitimacy of the enterprise (Reed, 2004; Geng et al., 2021). The higher consumers’ evaluation of cognitive legitimacy is, the higher their evaluation of the product (Bolton and Reed, 2004), and individuals with a higher risk propensity are more likely to seek additional information (Cho and Lee, 2006). We further anticipate that consumers with higher risk propensity are more likely to obtain positive cognition of the enterprise from the public. Positive cognition will improve cognitive legitimacy and increase the purchase intention of consumers (Li et al., 2014). Therefore, we hypothesize the following:

*Hypothesis 2*: Cognitive legitimacy mediates the relationship between risk propensity and purchase intention.

### Moderating Role of Perceived Benefit

Perceived benefit is a subjective evaluation formed by consumers through a comprehensive evaluation of the experience value of products and services. Perceived value refers to “the customer’s standard assessment of the utility of a product (or service) based completely on perceptions of what is acquired and what is given” (Jiang et al., 2018). In marketing, perceived benefit is initially defined as the subjective evaluation formed after the consumer weighs the perceived loss and the perceived profit of the product or service (Zeithaml, 1988). Perceived loss consists of all expenses confronted by the purchaser when purchasing, such as price, transportation, set up and maintenance, as properly as the threat of buying failure or unsatisfactory quality; perceived earnings refer to bodily property, carrier property and handy technical aid of the product in the course of product buy and use (Flint et al., 1997; Forlani et al., 2002).

On this basis, Woodruff and Gardial (1996) proposed that consumers’ perceived benefit of products would influence their purchase intention. Perceived benefit is the subjective psychological evaluation of consumers, which has an important impact on whether consumers can form purchase intentions. When the total perceived benefit obtained by consumers from products and services is higher than the total perceived cost, consumers believe that products have higher perceived benefits (Khalifa, 2004) and are more likely to meet consumers’ personal expectations, thus generating their purchase intention. Specifically, different consumers have different levels of risk propensity. When consumers perceive higher benefits, consumers’ expectations are more likely to be satisfied. Accordingly, consumers are more likely to have a positive evaluation of enterprises; that is, consumers form a higher cognitive legitimacy of enterprises.

*Hypothesis 3*: Perceived benefit moderates the relationship between risk propensity and cognitive legitimacy such that this relationship is stronger when perceived benefit is high rather than low.

### A Moderated Mediation Model

In this paper, we predict that perceived benefit moderates the relationship between risk propensity and cognitive legitimacy. Individuals are more likely to view the organization as legitimate when it provides products or services that meet their expectations, which corresponds to the favorable attitude of the public toward the organization, namely, cognitive legitimacy (Jahn et al., 2020). The higher the perceived benefit of a product or service, the higher the perception of value will be (Zeithaml, 1988). Thus, consumers generate a favorable attitude toward the enterprises and improve their cognitive legitimacy. In other words, consumers’ high perceived returns enhance the indirect relationship between risk propensity and purchase intention by moderating the mediating effect. Hence, by facilitating cognitive legitimacy, consumers with a high perceived benefit tend to activate their purchase predisposition with high risk propensity. To provide an explanation for the impact of risk propensity (independent variable) on purchase intention (dependent variable), we propose a moderated mediation model (i.e., the first-stage moderation). Based on the above analysis, we hypothesize the following:

*Hypothesis 4*: Perceived benefit moderates the mediating effect of cognitive legitimacy on the relationship between risk propensity and purchase intention such that the mediated effect of risk propensity on purchase intention through cognitive legitimacy is stronger when perceived benefit is high.

## Materials and Methods

### Participants and Procedures

To examine our hypotheses, we adopted the online survey method to collect data from March to April 2019 in Shanghai, China. Following snowball sampling procedures, the authors distributed survey links to 30 participants, and then these participants suggested that their relatives or friends respond to the survey. We assure respondents that their information is anonymous and confidential, and all data collected will be used for research purposes only. We invited consumers to reply to our survey, which assessed their risk propensity, purchase intention, perceived benefits, cognitive legitimacy and demographic variables, and rewarded participants with a $1 for filling out the questionnaire. After eliminating mismatched respondents, we obtained a final sample of 315 consumers, for a response rate of 85.6%. There were 111 males and 204 females, and the average age of the participants was 25.45 (SD = 7.11). In relation to income, the average income of the respondents was 2,320 yuan (SD = 1.58).

### Measures

With the survey items, all respondents were asked to complete a five-point Likert scale from 1 (strongly disagree) to 5 (strongly agree). To ensure the semantic and conceptual equivalence of devices expressed in distinctive languages, each item of each construct was subject to the back-translation procedure recommended by Brislin (1986).

#### Risk Propensity

Risk propensity was measured using the five-item scale from Cui et al. (2016). Participants answered items according to a Likert scale ranging from 1 (strongly disagree) to 5 (strongly agree). One of the sample items was “I have the ability to deal with risk.” The Cronbach’s *ɑ* coefficient was 0.783.

#### Cognitive Legitimacy

We used Pollack et al.’s (2012) five items to adapt to our research context based on the conceptualization of cognitive legitimacy. The scale was composed of five items to estimate the respondents’ recognition of enterprise behaviors. Participants were required to reply to questions on a 5-point Likert scale from 1 (strongly opposed) to 5 (strongly agreed). The Cronbach’s *ɑ* coefficient was 0.893.

#### Perceived Benefit

Five items from Kim et al. (2008) were used to appraise perceived benefit. Participants were required to rate on a 5-point scale from 1 (strongly opposed) to 5 (strongly agreed), such as “I think using this product is convenient.” The Cronbach’s *ɑ* for this scale was 0.837.

#### Purchase Intention

We rated participants’ purchase intention of the innovative product using a scale of 1 (strongly disagree) to 5 (strongly agree) from Hong et al. (2017). The 5-point Likert scale consists of four items, such as “I will frequently use the smartwatch in the future.” The Cronbach’s *ɑ* coefficient was 0.909.

#### Control Variables

A number of the subordinates’ demographic variables were managed to minimize the impacts of exogenous variables, including age, gender, and income. Previous research has proven that such variables are associated with risk propensity (Wang et al., 2009) and purchase behavior (Powell and Ansic, 1997; Bakshi, 2012).

### Measurement Models

A series of confirmatory factor analyses (CFAs) were conducted to examine the distinctiveness of the study’s variables based on the comparative fit index (CFI), the nonnormed fit index (NNFI), and the standardized root-mean-square residual (SRMR; Hu and Bentler, 1999). In this study, we developed four models, including a null model (*M*_0_); baseline four-factor model (*M*_1_); a combination of risk propensity and cognitive legitimacy (*M*_2_); a model combining three predictors of risk propensity, cognitive legitimacy and perceived benefit to evaluate their discrepancy (*M*_3_); and devised the last model to test whether the four constructs represent a single dimension (*M*_4_). Table 1 suggests that the basic CFA consequences demonstrated that the hypothesized four-factor model greatly enhanced the information (*χ*2 = 376.226, *df* = 113, CFI = 0.916, NNFI = 0.899, SRMR = 0.065). This four-factor dimension model additionally geared up the facts higher than different alternate dimension models. These CFA results indicate that the learning variables can be used in subsequent analyses.

**Table 1:** Confirmatory factor analyses of the measurement models.

Model specifications	*χ* ^2^	*df*	Δ*χ*^2^	CFI	NNFI	SRMR
Null model (*M*_0_)	3270.960	136	–	–	–	–
Baseline four-factor model (*M*_1_)	376.226	113	–	0.916	0.899	0.065
PR and CL are combined (*M*_2_)	568.802	116	192.576^**^	0.856	0.831	0.072
Three predictors (PR + CL + PB) are combined (*M*_3_)	830.277	118	454.051^**^	0.773	0.738	0.095
Four constructs represent a single dimension (*M*_4_)	1006.473	119	630.247^**^	0.717	0.676	0.099

*N* = 315. RP, risk propensity; CL, cognitive legitimacy; PB, perceived benefit. Δ*χ*^2^ is the change in *χ*^2^ compared with the baseline model.

^**^p < 0.01.

## Results

### Descriptive Statistics

Table 2 suggests the means, standard deviations and correlations among the variables. As shown in Table 2, risk propensity was positively related to cognitive legitimacy (*r* = 0.43, *p* < 0.01), perceived benefit (*r* = 0.31, *p* < 0.01) and consumers’ purchase intention (*r* = 0.46, *p* < 0.01). Cognitive legitimacy was once positively associated with perceived benefit (*r* = 0.51, *p* < 0.01) and consumers’ purchase intention (*r* = 0.71, *p* < 0.01). In addition, the correlation between consumers’ perceived benefit and consumers’ purchase intention was positively significant (*r* = 0.52, *p* < 0.01). In addition, control variables such as age, gender and income have a significant effect on the explained variables, and we control for three variables to eliminate the influence on the other variables. In brief, these results provide a preliminary basis for our hypotheses.

**Table 2:** Descriptive statistics, correlations and reliabilities.

	Mean	SD	1	2	3	4	5	6	7	8
1. Risk propensity	3.39	0.83	–								
2. Cognitive legitimacy	3.49	0.83	−0.43^**^	–							
3. Perceived benefit	3.80	0.79	0.31^**^	0.51^**^	–						
4. Continuance intention	3.46	0.98	0.46^**^	0.71^**^	0.52^**^	–					
5. Age	25.45	7.11	0.11^*^	0.16^**^	0.65	0.10	–				
6. Gender	1.65	0.48	−0.30^**^	−0.12^*^	−0.14^*^	−0.19^**^	−0.08	–			
7. Income	2.32	1.58	0.21^**^	0.15^*^	0.17^**^	0.24	0.42^**^	−0.21^*^	–		

*N* = 315. Gender: 1 = male, 2 = female.

* *p* < 0.05;

** *p* < 0.01.

### Hypotheses Testing

To further test our hypothesis, we conducted a series of hierarchical multiple regression analyses. The final result is shown in Table 3. In Model 2, consumers’ risk propensity was positively related to their purchase intention (*β* = 0.416, *p* < 0.001). Thus, Hypothesis 1 is supported.

**Table 3:** Results of mixed model analysis for the hypothesized relationships.

Variables	Continuance intention				Cognitive legitimacy			
	Model 1	Model 2	Model 3	Model 4	Model 5	Model 6	Model 7	Model 8
Control variables								
Age	−0.007	−0.021	−0.086	−0.085^*^	0.114	0.100	0.107^*^	0.108^*^
Gender	−0.142^*^	−0.031	−0.074	−0.040	−0.0988	0.015	0.027	0.028
Income	0.215^**^	0.157^**^	0.160^***^	0.144^**^	0.079	0.021	−0.028	−0.040
Independent variable								
Risk propensity		0.416^***^		0.147^**^		0.422^***^	0.307^***^	0.306^***^
Mediator								
Cognitive legitimacy			0.695^***^	0.638^***^				
Moderator								
Perceived benefit							0.415^***^	0.436^***^
Interaction term								
Risk propensity × Perceived benefit								0.117^*^
*R* ^2^	0.078	0.231	0.541	0.557	0.042	0.200	0.353	0.366
Δ*R*^2^	0.069	0.221	0.535	0.550	0.032	0.189	0.342	0.353
*F*	8.674^***^	23.181^***^	90.643^***^	77.127^***^	4.473^**^	19.192^***^	33.441^***^	29.424^***^

*N* = 315. Risk propensity, perceived benefit and their interaction were centered prior to analysis.

* *p* < 0.05;

** *p* < 0.01;

*** *p* < 0.001.

Hypothesis 2 proposed that consumers’ cognitive legitimacy mediates the relationship between risk propensity and purchase intention. First, we adopted the test proposed by Baron and Kenny (1986). Model 6 revealed consumers’ risk propensity to be positively related to the purchase intention of innovative products (*β* = 0.416, *p* < 0.001). In line with the results of Model 3, cognitive legitimacy was positively associated with purchase intention (*β* = 0.695, *p* < 0.001). Moreover, the coefficient of risk propensity for the purchase intention of the innovative product decreased from 0.416 to 0.147, showing that cognitive legitimacy plays a partial mediating role in the relationship between risk propensity and purchase intention. Taking these results into consideration, Hypothesis 2 is supported.

**Figure 2:**
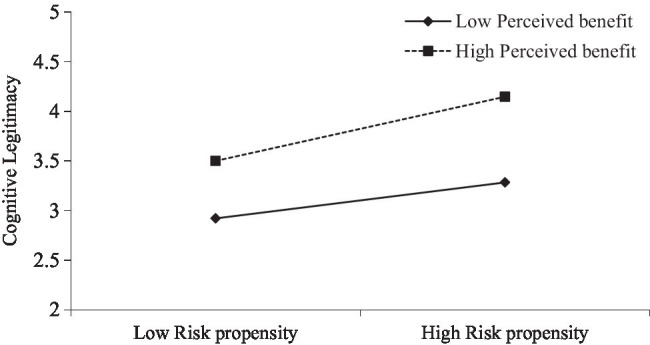
The moderating effect of perceived benefit on risk propensity for cognitive legitimacy.

The variance inflation factor (VIF) score of each variable (ranging from 1.07 to 2.20) indicated that multicollinearity was not a problem. Model 4 demonstrated that interaction items significantly positively predicted consumer cognitive legitimacy (*β* = 0.117, *p* < 0.05), suggesting that perceived benefit moderated the relationship between risk propensity and cognitive legitimacy. As shown in Figure 2, the relationship between risk propensity and cognitive legitimacy is stronger when perceived benefit is high rather than low. Thus, Hypothesis 3 is supported.

Hypothesis 4 predicted a first-stage moderated mediation; that is, consumers’ perceived benefit moderates the indirect relationship between risk propensity and purchase intention through cognitive legitimacy. We distinguish the level of perceived benefit in light of the values for moderators being the mean and plus/minus one SD from the mean. Table 4 shows the indirect effect of risk propensity on purchase intention through cognitive legitimacy at three levels of moderator. The results showed that for consumers with high perceived benefit, the intervals of the bootstrap test were (0.17, 0.41). For those with low perceived benefit, the mediating effect of cognitive legitimacy was significant, and the interval was (0.05, 0.28), containing zero. Based on the text, Hypothesis 4 is supported.

**Table 4:** Conditional indirect effect at specific values of power distance.

	Effect	SE	Boot LLCI	Boot ULCI
Low PB, RP-CL-CITU	0.16	0.06	0.05	0.28
Medium PB, RP-CL-CITU	0.23	0.05	0.13	0.32
High PB, RP-CL-CITU	0.29	0.06	0.17	0.41

*N* = 315. RP, risk propensity; CL, cognitive legitimacy; CITU, continuance use intention; PB, perceived benefit.

## Discussion

This study conducted a moderated mediation model to make a thorough inquiry into the relationship between risk propensity and purchase intention. The results show that individuals with high risk propensity are more inclined to attempt innovative products and that the relationship is mediated by consumers’ cognitive legitimacy. Moreover, we find that impact of risk propensity on purchase intention through cognitive legitimacy is moderated by consumers’ perceived benefit. For consumers with high perceived benefits, the higher their risk propensity is, the more cognitive legitimacy they are to enterprise behaviors. As a result, they are more likely to generate purchase intention. In contrast, for consumers with low perceived benefit, risk propensity cannot promote their cognitive legitimacy and purchase intention.

### Theoretical Implications

Our study makes three theoretical contributions. First, we contribute to the literature on marketing management by reviewing the relevant literature on factors influencing consumers’ purchase intention in Shanghai, China. In the international business literature, research on the risk propensity of consumers is scarce (Massad and Reardon, 1996; Sharma et al., 2009; Kusumasondjaja, 2015). With an increasing number of technology-based enterprises dedicated to innovative product design and consumers becoming more selective about products, the demand for innovative products is growing rapidly (Hajli et al., 2017).

In addition, our research adds to emerging research on how risk propensity shapes consumers’ purchase intention. Although studies have shown that perceived risk has a significant positive impact on consumers’ purchase behavior (Pelaez et al., 2019), the underlying mechanism is still unclear. This study introduces cognitive legitimacy into our theoretical model according to the concept drawn from the research on social enterprises. From the perspective of TPB (Fishbein and Ajzen, 1977), our results demonstrate the mediating effect of cognitive legitimacy on the relationship between risk propensity and purchase intention. This suggests that risk propensity may act as a key point to cope with nurturing consumers’ positive attitude toward enterprise behaviors. On one hand, the mediating model enriches the theoretical research on individual characteristics; on the other hand, it contributes to helping managers better understand the attitude of consumers toward enterprise behaviors. Moreover, we believe that the notion of legitimacy in the business literature deserves further exploration.

### Practical Implications

Our research contributes to technology-based enterprises toward the implementation of precision marketing management. First, our research can help senior managers understand how the risk propensity of consumers promotes purchase intention. The results reveal that consumers with higher risk propensity are more willing to try innovative products. Considering the important role of innovative products in the business environment (Cahen et al., 2016), it is critical for technology-based enterprises to improve the market share of their products. Being aware of the importance of risk propensity and making different sales guidance for consumers with different risk propensities is the key for enterprises to survive in the fierce business environment. Technology-based enterprises should accurately analyze the risk propensity of their target consumers with big data. For example, it is important for enterprises to guide consumers who have experience in purchasing innovative products with the technical properties of products. Moreover, enterprises could launch new word-of-mouth networks by providing opinion leaders with the opportunity to experience the latest products first.

Second, our study reveals that cognitive legitimacy plays a crucial role in improving consumers’ purchase intention. This finding calls attention to us that enterprises should show solicitude for consumers’ attitudes toward firm behaviors. In a competitive market, high cognitive legitimacy contributes to both the enterprise and its consumers. In addition, creating a high legitimacy would help attract consumers and gain investment (Delmar and Shane, 2004). Specifically, enterprises can actively undertake social responsibilities to establish a good public image. As a result, the public feels that their values are similar to those of enterprises to help enterprises obtain moral legitimacy. On the other hand, by guiding the media, enterprises can guide public opinion and communicate with the public and other stakeholders with the help of the media to gradually penetrate public life and obtain cognitive legitimacy.

### Limitations and Future Research Directions

Our research has several limitations. First, the data used in this study are cross-sectional in nature, which has certain limitations in the verification of causality. To obtain more vigorous conclusions, future studies can be further verified by using longitudinal data. Second, this study regards purchase intention as the process of behavioral intention and does not focus on specific actions after the formation of intention. That is, whether there is real purchase behavior is unclear. Therefore, future research can incorporate subsequent purchase behaviors into the research. Third, the effect of gender on risk propensity was not considered in this study. Studies have proven that males and females fluctuate in their risk preferences (Powell and Ansic, 1997) and that males have less pessimistic risk estimates than females (Lerner et al., 2003), so we speculate that males are more willing to try innovative products. In the future, gender could be included in the study of risk propensity and purchase intention.

Finally, we discuss the perceived benefits only between risk propensity and cognitive legitimacy as well as the positive regulatory role in the model. In the process of purchase intentions form, there may be other variables to moderate the relationship, such as negative propaganda – potential consumers perceived distrust, due to the excessive publicity of innovative products. Emerging business models, such as online celebrities selling goods, may also have a complex impact on the cognitive legitimacy of enterprises and indirectly affect consumers’ willingness. The emergence of new business models provides a new direction for future research.

## Data Availability Statement

The raw data supporting the conclusions of this article will be made available by the authors, without undue reservation.

## Ethics Statement

Ethical review and approval was not required for the study on human participants in accordance with the local legislation and institutional requirements. The patients/participants provided their written informed consent to participate in this study.

## Author Contributions

JL developed the theoretical framework and worked on the literature review and manuscript writing. FG developed the theoretical framework and worked on data collection and analysis. ZY worked on the literature review and data collection. FG and JX worked on data analysis and manuscript writing. All authors contributed to the article and approved the submitted version.

## Funding

This research was funded by the Research and Innovation Team Project of Young Teachers in Shanghai International Studies University (“Research on Practices and Experiences of Grass-root City Governance in and out China”), Humanity and Social Science Foundation of Ministry of Education of China (21YJA630052), Qinglan Project of Jiangsu Province (2021), and Jiangsu Innovation and Entrepreneurship Talent Program (2021).

## Conflict of Interest

The authors declare that the research was conducted in the absence of any commercial or financial relationships that could be construed as a potential conflict of interest.

## Publisher’s Note

All claims expressed in this article are solely those of the authors and do not necessarily represent those of their affiliated organizations, or those of the publisher, the editors and the reviewers. Any product that may be evaluated in this article, or claim that may be made by its manufacturer, is not guaranteed or endorsed by the publisher.
